# Audit to Assess Melatonin Prescribing in Community CAMHS

**DOI:** 10.1192/bjo.2022.489

**Published:** 2022-06-20

**Authors:** Chirag Shroff

**Affiliations:** Alder Hey NHS Trust, Liverpool, United Kingdom

## Abstract

**Aims:**

Disordered sleep is common, affecting 20–30%of children aged 1–5 years and often continues later into childhood. Neurodevelopmental disorders and psychiatric comorbidities pose a greater risk. The audit aimed to determine whether clinical prescribing practice of melatonin in Burlington House, Sefton CAMHS reflected current NICE recommendations. NICE suggest that first-line treatments for children with sleep problems include good sleep hygiene and behavioural therapy (including sleep diary).

**Methods:**

Nice guideline CG170 provides guidance on Autism management. BNFC states that melatonin therapy should be reviewed every 6 months. Records of children currently prescribed modified release melatonin were checked to see if they met the inclusion criteria. Data were collected retrospectively from clinical case files and pharmacy records (December 2020- February 2021).

**Results:**

The results showed 18 young persons received melatonin for insomnia with ASD, 26 for insomnia without ASD, 3 for likely ASD and none for Smith Magenis syndrome. 36 received Specialist CAMHS review, 9 received Community Pediatrics review and 2 GP review. All patients received melatonin as per dose recommendations with 6 monthly reviews. Documentation on sleep hygiene was unclear.

**Conclusion:**

We concluded that Melatonin prescribing in community CAMHS tends to be high and discussion on sleep hygiene measures must be given importance.

